# Baseline characteristics of children with juvenile dermatomyositis enrolled in the first year of the new Childhood Arthritis and Rheumatology Research Alliance registry

**DOI:** 10.1186/s12969-022-00709-3

**Published:** 2022-07-19

**Authors:** Jessica Neely, Kaveh Ardalan, Adam Huber, Susan Kim, for the Childhood Arthritis and Rheumatology Research Alliance Investigators, N. Abel, K. Abulaban, A. Adams, M. Adams, R. Agbayani, J. Aiello, S. Akoghlanian, C. Alejandro, E. Allenspach, R. Alperin, M. Alpizar, G. Amarilyo, W. Ambler, E. Anderson, S. Ardoin, S. Armendariz, E. Baker, I. Balboni, S. Balevic, L. Ballenger, S. Ballinger, N. Balmuri, F. Barbar-Smiley, L. Barillas-Arias, M. Basiaga, K. Baszis, M. Becker, H. Bell-Brunson, E. Beltz, H. Benham, S. Benseler, W. Bernal, T. Beukelman, T. Bigley, B. Binstadt, C. Black, M. Blakley, J. Bohnsack, J. Boland, A. Boneparth, S. Bowman, C. Bracaglia, E. Brooks, M. Brothers, A. Brown, H. Brunner, M. Buckley, M. Buckley, H. Bukulmez, D. Bullock, B. Cameron, S. Canna, L. Cannon, P. Carper, V. Cartwright, E. Cassidy, L. Cerracchio, E. Chalom, J. Chang, A. Chang-Hoftman, V. Chauhan, P. Chira, T. Chinn, K. Chundru, H. Clairman, D. Co, A. Confair, H. Conlon, R. Connor, A. Cooper, J. Cooper, S. Cooper, C. Correll, R. Corvalan, D. Costanzo, R. Cron, L. Curiel-Duran, T. Curington, M. Curry, A. Dalrymple, A. Davis, C. Davis, C. Davis, T. Davis, F. De Benedetti, D. De Ranieri, J. Dean, F. Dedeoglu, M. DeGuzman, N. Delnay, V. Dempsey, E. DeSantis, T. Dickson, J. Dingle, B. Donaldson, E. Dorsey, S. Dover, J. Dowling, J. Drew, K. Driest, Q. Du, K. Duarte, D. Durkee, E. Duverger, J. Dvergsten, A. Eberhard, M. Eckert, K. Ede, B. Edelheit, C. Edens, C. Edens, Y. Edgerly, M. Elder, B. Ervin, S. Fadrhonc, C. Failing, D. Fair, M. Falcon, L. Favier, S. Federici, B. Feldman, J. Fennell, I. Ferguson, P. Ferguson, B. Ferreira, R. Ferrucho, K. Fields, T. Finkel, M. Fitzgerald, C. Fleming, O. Flynn, L. Fogel, E. Fox, M. Fox, L. Franco, M. Freeman, K. Fritz, S. Froese, R. Fuhlbrigge, J. Fuller, N. George, K. Gerhold, D. Gerstbacher, M. Gilbert, M. Gillispie-Taylor, E. Giverc, C. Godiwala, I. Goh, H. Goheer, D. Goldsmith, E. Gotschlich, A. Gotte, B. Gottlieb, C. Gracia, T. Graham, S. Grevich, T. Griffin, J. Griswold, A. Grom, M. Guevara, P. Guittar, M. Guzman, M. Hager, T. Hahn, O. Halyabar, E. Hammelev, M. Hance, A. Hanson, L. Harel, S. Haro, J. Harris, O. Harry, E. Hartigan, J. Hausmann, A. Hay, K. Hayward, J. Heiart, K. Hekl, L. Henderson, M. Henrickson, A. Hersh, K. Hickey, P. Hill, S. Hillyer, L. Hiraki, M. Hiskey, P. Hobday, C. Hoffart, M. Holland, M. Hollander, S. Hong, M. Horwitz, J. Hsu, A. Huber, J. Huggins, J. Hui-Yuen, C. Hung, J. Huntington, A. Huttenlocher, M. Ibarra, L. Imundo, C. Inman, A. Insalaco, A. Jackson, S. Jackson, K. James, G. Janow, J. Jaquith, S. Jared, N. Johnson, J. Jones, J. Jones, J. Jones, K. Jones, S. Jones, S. Joshi, L. Jung, C. Justice, A. Justiniano, N. Karan, K. Kaufman, A. Kemp, E. Kessler, U. Khalsa, B. Kienzle, S. Kim, Y. Kimura, D. Kingsbury, M. Kitcharoensakkul, T. Klausmeier, K. Klein, M. Klein-Gitelman, B. Kompelien, A. Kosikowski, L. Kovalick, J. Kracker, S. Kramer, C. Kremer, J. Lai, J. Lam, B. Lang, S. Lapidus, B. Lapin, A. Lasky, D. Latham, E. Lawson, R. Laxer, P. Lee, P. Lee, T. Lee, L. Lentini, M. Lerman, D. Levy, S. Li, S. Lieberman, L. Lim, C. Lin, N. Ling, M. Lingis, M. Lo, D. Lovell, D. Lowman, N. Luca, S. Lvovich, C. Madison, J. Madison, S. Magni Manzoni, B. Malla, J. Maller, M. Malloy, M. Mannion, C. Manos, L. Marques, A. Martyniuk, T. Mason, S. Mathus, L. McAllister, K. McCarthy, K. McConnell, E. McCormick, D. McCurdy, P. McCurdy Stokes, S. McGuire, I. McHale, A. McMonagle, C. McMullen-Jackson, E. Meidan, E. Mellins, E. Mendoza, R. Mercado, A. Merritt, L. Michalowski, P. Miettunen, M. Miller, D. Milojevic, E. Mirizio, E. Misajon, M. Mitchell, R. Modica, S. Mohan, K. Moore, L. Moorthy, S. Morgan, E. Morgan Dewitt, C. Moss, T. Moussa, V. Mruk, A. Murphy, E. Muscal, R. Nadler, B. Nahal, K. Nanda, N. Nasah, L. Nassi, S. Nativ, M. Natter, J. Neely, B. Nelson, L. Newhall, L. Ng, J. Nicholas, R. Nicolai, P. Nigrovic, J. Nocton, B. Nolan, E. Oberle, B. Obispo, B. O’Brien, T. O’Brien, O. Okeke, M. Oliver, J. Olson, K. O’Neil, K. Onel, A. Orandi, M. Orlando, S. Osei-Onomah, R. Oz, E. Pagano, A. Paller, N. Pan, S. Panupattanapong, M. Pardeo, J. Paredes, A. Parsons, J. Patel, K. Pentakota, P. Pepmueller, T. Pfeiffer, K. Phillippi, D. Pires Marafon, K. Phillippi, L. Ponder, R. Pooni, S. Prahalad, S. Pratt, S. Protopapas, B. Puplava, J. Quach, M. Quinlan-Waters, C. Rabinovich, S. Radhakrishna, J. Rafko, J. Raisian, A. Rakestraw, C. Ramirez, E. Ramsay, S. Ramsey, R. Randell, A. Reed, A. Reed, A. Reed, H. Reid, K. Remmel, A. Repp, A. Reyes, A. Richmond, M. Riebschleger, S. Ringold, M. Riordan, M. Riskalla, M. Ritter, R. Rivas-Chacon, A. Robinson, E. Rodela, M. Rodriquez, K. Rojas, T. Ronis, M. Rosenkranz, B. Rosolowski, H. Rothermel, D. Rothman, E. Roth-Wojcicki, K. Rouster-Stevens, T. Rubinstein, N. Ruth, N. Saad, S. Sabbagh, E. Sacco, R. Sadun, C. Sandborg, A. Sanni, L. Santiago, A. Sarkissian, S. Savani, L. Scalzi, L. Schanberg, S. Scharnhorst, K. Schikler, A. Schlefman, H. Schmeling, K. Schmidt, E. Schmitt, R. Schneider, K. Schollaert-Fitch, G. Schulert, T. Seay, C. Seper, J. Shalen, R. Sheets, A. Shelly, S. Shenoi, K. Shergill, J. Shirley, M. Shishov, C. Shivers, E. Silverman, N. Singer, V. Sivaraman, J. Sletten, A. Smith, C. Smith, J. Smith, J. Smith, E. Smitherman, J. Soep, M. Son, S. Spence, L. Spiegel, J. Spitznagle, R. Sran, H. Srinivasalu, H. Stapp, K. Steigerwald, Y. Sterba Rakovchik, S. Stern, A. Stevens, B. Stevens, R. Stevenson, K. Stewart, C. Stingl, J. Stokes, M. Stoll, E. Stringer, S. Sule, J. Sumner, R. Sundel, M. Sutter, R. Syed, G. Syverson, A. Szymanski, S. Taber, R. Tal, A. Tambralli, A. Taneja, T. Tanner, S. Tapani, G. Tarshish, S. Tarvin, L. Tate, A. Taxter, J. Taylor, M. Terry, M. Tesher, A. Thatayatikom, B. Thomas, K. Tiffany, T. Ting, A. Tipp, D. Toib, K. Torok, C. Toruner, H. Tory, M. Toth, S. Tse, V. Tubwell, M. Twilt, S. Uriguen, T. Valcarcel, H. Van Mater, L. Vannoy, C. Varghese, N. Vasquez, K. Vazzana, R. Vehe, K. Veiga, J. Velez, J. Verbsky, G. Vilar, N. Volpe, E. von Scheven, S. Vora, J. Wagner, L. Wagner-Weiner, D. Wahezi, H. Waite, J. Walker, H. Walters, T. Wampler Muskardin, L. Waqar, M. Waterfield, M. Watson, A. Watts, P. Weiser, J. Weiss, P. Weiss, E. Wershba, A. White, C. Williams, A. Wise, J. Woo, L. Woolnough, T. Wright, E. Wu, A. Yalcindag, M. Yee, E. Yen, R. Yeung, K. Yomogida, Q. Yu, R. Zapata, A. Zartoshti

**Affiliations:** 1 University of California San Francisco Medical Center, 550 16th Street, San Francisco, CA 94158 USA; 2 Duke University Medical Center, 2301 Erwin Rd, Durham, NC 27705 USA; 3 Division of Pediatric Rheumatology, IWK Health Centre and Dalhousie University, PO Box 9700, 5850-5980 University Ave, Halifax, Nova Scotia Canada

**Keywords:** Cohort studies, Pediatric rheumatology, Juvenile Dermatomyositis, Registry, Patient reported outcomes

## Abstract

**Background:**

To report baseline characteristics, patient reported outcomes and treatment of children with Juvenile Dermatomyositis (JDM) in the Childhood Arthritis and Rheumatology Research Alliance (CARRA) Registry.

**Methods:**

Children newly diagnosed with JDM were enrolled in the CARRA Registry from 41 pediatric rheumatology centers. Baseline patient demographics, disease characteristics, assessments, patient reported outcome and treatments were recorded.

**Results:**

In the first year, 119 JDM participants were enrolled. Most were female (63.4%), and white (72.3%) with a median diagnosis age 8.0 years (IQR 4.0–11.5), and median age of disease onset 7.0 years (IQR 3.5–7.5). They had characteristic rashes (92.4%), elevated muscle enzymes (83.2%), physician global score 4.0 (IQR 2.5–5.0) and manual muscle testing score 63.5 (IQR 51.0–75.0). Calcinosis (3.4%) and interstitial lung disease (< 1%) were uncommon. Myositis specific antibodies were measured and reported in nearly half of participants enrolled where anti-MJ followed by Anti-p155/140 were most common (11/49 and 7/53 respectively).

Childhood Health Assessment Questionnaire (CHAQ) results showed mild-moderate disability (median 0.750, IQR 0.030–1.875), as did patient/parent global assessments of disease activity (median 3, patient IQR: 1.75–5.25; parent IQR: 1–7). Patient Reported Outcomes Measurement Information System (PROMIS®) Pediatric Global Health 7 scores, Pain Interference, Physical Function scores for Mobility, and Upper Extremity Function were commonly worse than 95% of the general pediatric population.

**Conclusions:**

In its inaugural year, 119 JDM patients were successfully enrolled in participapte in the New CARRA Registy. This registry will provide the necessary foundation to advance clinical research to improve outcomes using traditional measures and patient reported outcomes. With the CARRA biorepository, this infrastructure will enable future translational research. Together, these efforts may aid in future clinical trials, including comparative effectiveness trials.

## Background

Juvenile dermatomyositis (JDM) is the most common inflammatory myopathy in children but it is nonetheless rare, with an estimated annual incidence of 2–4 in 1 million children in the United States [1]. JDM is a systemic autoimmune disease characterized by pathognomonic rashes and proximal muscle weakness which may also involve many organ systems including the heart, lungs and gastrointestinal tract. Outcomes for JDM before the 1960s were poor, with a mortality rate of nearly 30% [2]. With the advent of steroids and other immunomodulatory therapies, mortality has declined to approximately 2% [3–5] in North America and the United Kingdom. Despite these improvements in outcomes, the severity and disease course is highly variable and over 60% of patients with JDM continue to experience a chronic continuous or polycyclic disease course, so remission and cure are uncommon [6, 7]. Although functional outcomes for JDM have improved with therapy, the long-term outcomes of North American and European cohorts suggest that the majority of patients suffer from persistently active disease, and that nearly 60% of patients develop disease damage including chronic weakness, joint contractures, calcinosis, and lipodystrophy [6–9].

There are few clinical trials to inform optimal treatment approaches in JDM. Given the challenges of conducting clinical trials in such a rare disease, large observational cohort studies are vital to improving the care and outcomes of children with JDM through disease characterization, long-term monitoring, and comparative effectiveness studies.

To this end, following the success of the historical pilot registry known as the Childhood Arthritis and Rheumatology Research Alliance (CARRA) Legacy Registry [10], the New CARRA Registry [11] was initiated in 2015 and began enrollment of JDM patients in 2019. The New CARRA Registry includes prospective data collection to document the clinical course and medications used to treat childhood-onset rheumatic conditions which will facilitate long-term safety monitoring of the medications used to treat these conditions. In addition, the New CARRA Registry has several innovations to overcome some barriers in studying this rare condition. First, data collection forms, modeled on the international consensus core dataset for JDM [12], were standardized to facilitate harmonization with ongoing investigator-led studies and other cohorts and registries worldwide. In addition, to facilitate future comparative effectiveness analyses, the JDM Registry includes the previously developed consensus treatment plans for Moderate [13] and Skin-Predominant JDM [14]. The Registry is designed as a longitudinal inception cohort with data collection planned over a minimum of 10 years, which will abrogate the limitations encountered with cross-sectional data. The systematic collection of patient reported outcomes was also included in the data collection. Lastly, in order to bolster translational studies, standardized biosample collection was subsequently added to this registry.

Here, we describe the baseline patient demographics, disease characteristics, initial assessments, patient/parent-reported outcomes and treatments for 119 children with JDM enrolled in the current registry in the first year.

## Methods

### Study population and inclusion criteria

The New CARRA Registry [11] began enrollment of JDM participants in January 2019 at 71 participating sites across North America. Participants were eligible for enrollment if they were < 18 years of age at disease onset and diagnosed with JDM based on clinical expertise of the treating rheumatologist and Bohan and Peter’s criteria [15, 16]. To be eligible for enrollment, participants needed to have been diagnosed no more than 6 months prior to enrollment and treated with systemic therapy for no more than 12 weeks. The New CARRA Registry was approved by Duke University Institutional Review Board (Pro00054616) and each participating site obtained local IRB approval. Data collection complies with the Declaration of Helsinki. Eligible patients and their families were approached by the local investigators, and written informed consent was obtained from participants age 18 years or older, or from a parent or legal guardian of younger participants who had given their assent.

### Data collection

Assessments were performed by medical providers and participants/guardians using standardized case report forms. At the baseline visit, demographic variables, including age, sex, race, insurance, household income, and parental educational level, along with medical history regarding comorbid conditions and relevant family history in first degree family members, was recorded. Information regarding diagnostic physical exam findings, diagnostic tests, and laboratory results was also recorded.

### Disease activity indices

Clinicians also documented the presence or absence of extramuscular disease activity for constitutional, skeletal, gastrointestinal, pulmonary, and cardiovascular disease and provided a global visual analog score (VAS) ranging from 0 to 10 for each domain. VAS scores were also recorded for muscle, skin and global domains [17]. Results of two graded muscle strength evaluations, the Manual Muscle Testing 8 (MMT8) Scale and the Childhood Myositis Assessment Scale (CMAS), were also recorded to assess muscle strength and endurance: data recorded for participants < 5 years of age were excluded for CMAS and MMT8 due to limitations in validity of these measures in the younger patients [18]. The binary version of the Cutaneous Activity Tool (CAT) and Cutaneous Dermatomyositis Area and Severity Instrument (CDASI) were scored to assess skin disease activity and damage [19].

### Disease damage indices

The myositis damage index (MDI) and presence of calcinosis were recorded to assess disease damage at the time of enrollment [20].

### Patient reported outcomes

Standardized questionnaires were recorded from the parent of the subject or the subject, depending on the age of the patient. These assessments included patient or parent global VAS score (0–10), the Childhood Health Assessment Questionnaire (CHAQ), Faces Pain Scale (0–10) from all participants, and Patient Reported Outcomes Measurement Information System (PROMIS**®**) measures in the domains of pain intensity, pain interference, global health (encompassing physical and mental health and quality of life), and physical function (i.e. mobility and upper extremity function) [20–22] for participants 3 years of age or older. CHAQ respondent (i.e. patient or parent) was not documented, so CHAQ data were pooled.

### Medications

**Fig. 1:**
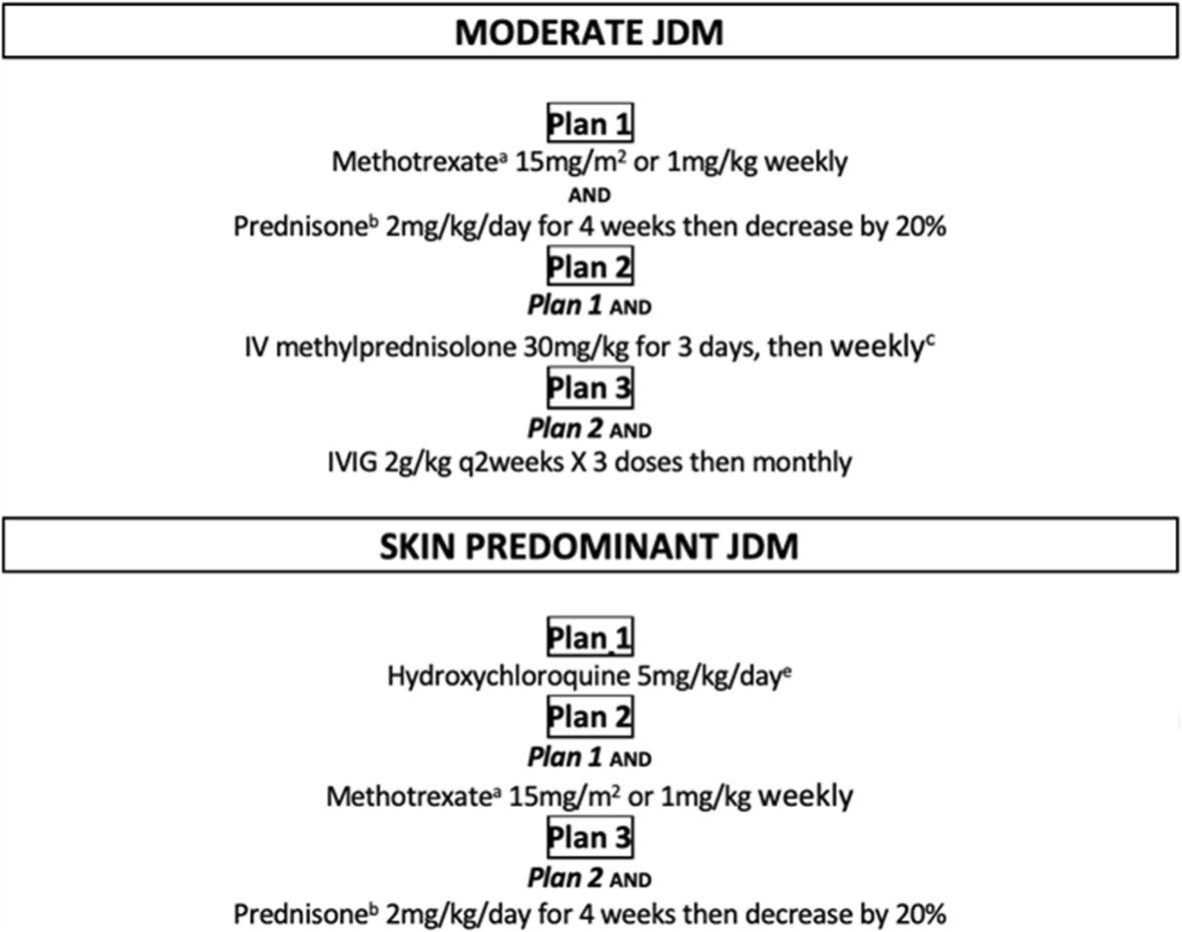
Published consensus treatment plans for new onset JDM patients with moderate, skin predominant and skin resistant disease

Prescribed medications were recorded at the time of baseline enrollment. History of medication use was recorded, along with whether the participant was being treated according to a CARRA consensus treatment plan (CTP), namely either the CTP for moderate JDM or the CTP for skin-predominant JDM. For each CTP, investigators also recorded which one of the three treatment approaches shown in Fig. 1 was selected.

### Statistics

Descriptive statistics were applied to evaluate the baseline characteristics of this cohort. For variables with a normal distribution, the mean and standard deviation was calculated, and for those with a non-normal distribution, the median and interquartile range was calculated. To perform a sensitivity analysis to determine contributing factors to missing MMT-8 and CMAS measurements, the mean age, sex and physician global scores between those with and without each measurement was compared using a t-test. To perform a stratified analysis to determine if participants enrolled to the registry prior to treatment had higher disease activity levels than those enrolled after treatment was started, the median physician VAS scores for global, muscle and skin domains were compared using a Wilcoxon test.

## Results

One hundred nineteen patients with JDM meeting eligibility criteria were enrolled in the New CARRA Registry between January 2019 and December 2019 from 41 out of 71 sites across North America. About half of all participants, 60/119 (50.4%), were treatment-naïve at the time of enrollment, and the remaining participants were enrolled within the 12-week treatment window. Sixty-three percent of participants were female (*n* = 76), with a median age at diagnosis of 8.0 years (IQR 4.0–11.5), and median age at disease onset of 7.0 years (IQR 3.5–7.5) (Table 1). Seventy-two percent of participants were white (*n* = 86) and 18.5% (*n* = 22) of participants reported Hispanic, Latino, or Spanish descent (Table 1).

**Table 1:** Demographics and diagnostic features (*N* = 119)

Age at diagnosis in years, median (IQR)	8 (4.0–11.5)
Age at disease onset in years, median (IQR)	7 (3.5–7.5)
Time to diagnosis in months, median (IQR)	3 (1–6.5)
Female, N (%)	76 (63.4)
Race or Ethnicity^a^, N (%)	
White	86 (72.3)
Hispanic, Latino, or Spanish origin	22 (18.5)
Black, African American, African, or Afro-Caribbean	9(7.6)
Asian	7(5.9)
Native American, American Indian or Alaskan Native	3 (2.5)
Middle Eastern	3 (2.5)
Unknown^b^	3 (2.5)
Other^c^	4 (3.4)
Concomitant Medical History, N (%)^d^	22 (16.8)
Family History of Autoimmunity, N (%)^e^	25 (21)
Skin Predominant JDM, N (%)	38 (31.9)
History of, N (%)	
Proximal Muscle Weakness	86 (72.3)
Rash (Heliotrope or Gottron’s)	110 (92.4)
Elevated muscle enzymes	99 (83.2)
EMG performed	4 (3.4)
Muscle Biopsy performed	19 (16)
MRI performed	81 (68.1)
Autoantibodies, proportion^f^	
ANA	75/96 (78.1%)
Myositis-specific antibodies	
Anti-MJ/NXP2	11/49 (22.4%)
Anti-p155/140/TIF1-𝛄	7/53 (13.2%)
Anti-Mi2	6/55 (10.9%)
Anti-MDA5	4/51 (7.8%)
Anti-Jo1	2/67 (3.0%)
Myositis-associated antibodies	
Anti-PM-Scl	3/43 (7.0%)
Anti-Smith	1/56 (1.7%)

^a^Some participants reported more than one Race and/or Ethnicity

^b^Patient/guardian chose “other” and/or “not any of the Races”

^c^Patient/guardian chose “prefer not to answer” and/or did not provide a responsed

^d^Twenty-two of the enrolled participants had a history of at least one other medical condition: 5 participants had a history of asthma, 3 autoimmune thyroid disease, 1 celiac disease, 2 other autoimmune disease, and 12 other major or or acquired disease. One participant had a history of multiple autoimmune conditions including thyroid, celiac, and other autoimmune disease in addition to JDM

^e^Twenty-five participants had a history of autoimmunity in first-degree family members, and 5 of these participants had a family history of more than one autoimmune condition. The most common condition was psoriasis in 9 family members, followed by rheumatoid arthritis in 4, systemic lupus erythematosus in 3, inflammatory bowel disease in 3, autoimmune thyroid disease in 3, ankylosing spondylitis in 2, celiac disease in 2, juvenile arthritis in 1, Sjogren’s disease in 1, and other autoimmune disease in 4

^f^Proportion of patients with a positive test of the total number tested

### Medical and family history

Twenty-two of the enrolled participants had a history of at least one other medical condition and 25 participants had a history of autoimmunity in first-degree family members, and 5 of these participants had a family history of more than one autoimmune condition (Table 1).

### Diagnostic features

Participants with characteristic rashes (Gottron’s papules/rash or heliotrope rash) were reported in 110/119 (92.4%), and symmetric proximal muscle weakness in 86/119 (72.3%), and elevated muscle enzymes in 99/119 (83.2%) (Table 1). MRI was the most commonly performed diagnostic test in 81/119 (68.1%) of participants, whereas diagnostic muscle biopsy and EMG were less commonly performed in 19/119 (16%) and 4/119 (3.4%), respectively, indicating that invasive testing was rarely performed (Table 1).

### Autoantibodies

Of participants tested, 75/96 (78.1%) had a positive ANA. Approximately half of enrolled participants had myositis-specific antibody (MSA) testing from various laboratories, with anti-NXP2 (aka MJ) being the most common antibody identified in 11/49 (22.4%) followed by anti-TIF1-𝛄 (aka p155/140) in 7/53 (13.2%), anti-Mi2 in 6/55 (10.9%), anti-MDA5 in 4/51 (7.8%), and anti-Jo1 in 2/67 (3.0%) of participants tested (Table 1). No participants had positive anti-SRP or anti-HMGCR antibodies. Four of 119 participants had multiple MSA positivity, and were removed from this autoantibody summary due to concern for errors in reporting since it is rare for patients to have multiple MSAs. Myositis-associated antibodies (MAA) were reported positive for anti-PM-Scl 3/43 (7.0%) and anti-Smith 1/56 (1.8%). The were no reported positive MAAs for the remaining antibodies including anti-Ro, anti-La, or anti-RNP antibodies.

### Clinical features

The median physician global VAS at baseline was 4.0 [IQR 2.5–5.0]. The overall median physician global VAS for skin disease was 2.0 [IQR 1.0–4.0] with Gottron’s papules/rash being the most common rash present during the enrollment visit reported in 75.6%, followed by malar erythema in 65.5%, periungal capillary loop changes in 58.8%, and heliotrope rash in 53.8% (Table 2). Cutaneous ulcerations were present in 10.9% of participants and extensive cutaneous erythema in 14.3%. No participants had panniculitis. The median cutaneous activity score tool was 4.0 (IQR 2.0–5.0). CDASI activity score was performed in 52 participants who had a median score of 5.0 [IQR 3.0–10.0] (Table 2). Calcinosis was rare at disease presentation in this cohort occurring in only 4 participants. These 4 participants who developed calcinosis had a median duration of symptoms of 12 months (IQR 8–18 months) compared to 3 months (IQR 1–6 months) in those who did not develop calcinosis. However, because of the variation in duration of symptoms and few number of patients (*N* = 4), this did not meet statistical significance.

**Table 2:** Clinical disease features, *N* = 119^a^

Muscle Enzyme Elevation, N (%)	99 (83.2)
MMT8^b^, median (IQR)	63.5 (51.0–75.0), *N* = 32
CMAS^c^, median (IQR)	43.5 (30.5–51.0), *N* = 34
Skin Manifestations, N (%)	
Gottron’s papules or sign	90 (75.6)
Malar or facial erythema	78 (65.5)
Periungal capillary loop changes	70 (58.8)
Heliotrope Rash	64 (53.8)
Linear Erythema	33 (27.7)
Cuticular overgrowth	24 (20.2)
Non-sun exposed erythema	23 (19.3)
Extensive cutaneous erythema	17 (14.3)
Shawl Sign	13 (10.9)
Cutaneous Ulceration	13 (10.9)
Subcutaneous edema	11 (9.2)
V sign	10 (8.4)
Mucus membrane lesions	6 (5)
Livedo reticularis	6 (5)
Mechanic’s hands	6 (5)
Alopecia	4 (3.4)
CAT^d^ Score, median (IQR)	4.0 (2.0–5.0)
CDASI^e^ Activity Score, median (IQR)	5.0 (3.0–10.0), *N* = 52
Calcinosis, N (%)	4 (3.4)
Overlap features, N (%)	
Raynaud phenomenon	8 (5.0)
Sclerodactyly	5 (3.1)
Constitutional symptoms, N (%)	
Fever	13 (10.9)
Weight loss	22 (18.5)
Fatigue	71 (59.7)
Arthritis, N (%)	33 (27.7)
Gastrointestinal symptoms, N (%)	
Abdominal Pain	6 (5)
Dysphagia	21 (17.6)
Pulmonary symptoms, N (%)	
Interstitial lung disease	1 (0.8)
Dysphonia	14 (11.8)
Cardiovascular involvement, N (%)	1 (0.8)
Physician Global Assessment, median (IQR)	4.0 (2.5–5.0), *N* = 106
Physician Global Muscle Disease Activity, median (IQR)	3.0 (1.0–5.0), *N* = 78
Physician Global Skin Disease Activity, median (IQR)	2.0 (1.0–4.0), *N* = 71
Physician Global Extramuscular Disease activity, median (IQR)	0.25 (0.0–3.25), *N* = 72
Constitutional	2.0 (0.0–2.0), *N* = 84
Skeletal	0.0 (0.0–2.0), *N* = 88
Gastrointestinal	0.0 (0.0–0.4), *N* = 90
Pulmonary	0.0 (0.0–0.0), *N* = 89
Cardiovascular	0.0 (0.0–0.0), *N* = 88

^a^*N* = 119 unless indicated in the table where missing data reduced the total number of patients analyzed

^b^Manual-Muscle Testing 8

^c^Childhood Myositis Assessment Scale

^d^Cutaneous Activity Tool

^e^Cutaneous Dermatomyositis Area and Severity Instrument

The median physician global VAS for muscle disease was 3.0 [IQR 1.0–5.0] and muscle enzymes were elevated in 83.2%. MMT8 scores were available for 36 participants (30%), however, 4 of these participants were < 5 yrs. of age. For 32 participants age 5 and up with reported scores, the median MMT8 score was 63.5 [IQR 51.0–75.0] indicating moderate weakness. CMAS was performed in 38 participants (32%), 4 of whom were < 5 yrs. of age. For 34 participants age 5 and up, the median CMAS score was 43.5 [IQR 30.5–51.0] (Table 2). These assessments were not performed in the majority of participants. A sensitivity analysis comparing the age, sex and physician global VAS score between participants with available MMT8 and CMAS scores and those without available scores identified that those with missing scores were significantly younger: mean age 7.4 years compared to 9.4 years for MMT-8 (*p* = 0.027) and mean age 7.2 compared to 9.7 years for CMAS (*p* = 0.01). Likewise, 31/83 (37%) and 31/81 (38%) of participants with missing MMT-8 and CMAS scores, respectively, were less than age 5, indicating that young age is, in part, contributing to absence of reporting of for these measures. Organ involvement beyond skin and muscle disease was rare in this cohort (Table 2).

A stratified subanalysis between treatment-naïve and treated participants showed significantly higher median physician global scores in treatment-naive compared to the treated group: 4.75 [3.00, 5.00] and 3.00 [2.00, 5.00], respectively (*p* < 0.014). Physician global VAS for skin in treatment-naïve compared to treated participants was 3.00 (1.12–5.00) versus 2.00 (1.00–3.00), (*p* = 0.097). Patient/parent global VAS in treatment-naïve compared to treated participants was 3.00 (2.00–6.00) versus 2.00 (1.00–5.00), (*p* = 0.057).

### Treatment

Half of all participants, 60/119 (50.4%), were treatment-naïve at enrollment, and the remaining participants were enrolled within the 12-week treatment window. Approximately half of participants (64/119, 54.6%), were treated according to a CTP at the baseline visit. The majority (51/64, 80%), were treated according to a Moderate JDM CTP and 13 participants were treated according to a Skin-Predominant CTP (Fig. 1). Of those treated by the Moderate JDM CTP, there was a relatively equal distribution across the three treatments arms with 14 participants treated according to Plan A (IV + PO steroids + MTX), 21 treated according to Plan B (IV + PO steroids + MTX + IVIG), and 16 treated according to Plan C (PO steroids + MTX). Of those treated by the Skin-Predominant CTP, 9 participants were treated according to Plan C (HCQ + MTX + PO steroids), 2 according to Plan A (HCQ monotherapy) and 2 according to Plan B (HCQ + MTX).

### Damage

Four participants (3.4%) had calcinosis at the baseline visit indicating this complication is rare at disease presentation in this cohort. Damage assessed by the Myositis Damage Index was also rare with only 7 participants with non-zero physician global damage VAS scores. Of these 7 participants, the median physican global damage VAS was 3.0 (IQR 1.0–3.5), and the most commonly reported damage was limited to the muscle and skin as follows: clinically identified muscle atrophy (*n* = 3), muscle dysfunction defined as a decrease in aeurobic capacity (*n* = 3), poikiloderma (*n* = 3), and lipoatrophy/lipodystrophy (*n* = 2). Sclerodactyly (*n* = 1), depressed scar/cutaneous atrophy (*n* = 1), muscle weakness not attributable to active muscle disease (*n* = 1), gastrointestinal dysmotility (*n* = 1), and persistent dysphagia (*n* = 1) were also reported.

### Patient/parent-reported outcomes

Patient and parent global assessments of disease activity were rated similarly (Table 3), with median scores of 3 for both groups of respondents (patient IQR: 1.8–5.3; parent IQR: 1–7). PROMIS Pediatric Global Health 7 (PGH7) patient and parent scores were generally scored similarly, with median score (IQR) 38.8 (33.6–42.1) for participants and 34.6 (39.4–37.9) for parents, consistent with worse health than the general pediatric population; 39.6 and 52.9% of patient and parent PGH7 scores were consistent with worse general health than 95% of the pediatric population [22].

**Table 3:** Patient/parent-reported outcome measures

Global Assessments:	Median (IQR)	High symptom/low function scorers, n (%)^**a**^
Global Assessment of Disease Activity (patient), *n* = 48	3.0 (1.8–5.3)	–
Global Assessment of Disease Activity (parent), *n* = 33	3.0 (1.0–7.0)	–
PROMIS Pediatric Global Health 7 (patient), *n* = 48	38.8 (33.6–42.1)	19 (39.6)
PROMIS Pediatric Global Health 7 (parent), *n* = 34	34.6 (29.4–37.9)	18 (52.9)
**Physical Function:**		
CHAQ^b^, *n* = 102	0.750 (0.030–1.875)	–
PROMIS^c^Mobility (patient), *n* = 48	36.9 (32.9–48.4)	19 (39.6)
PROMIS Mobility (parent), *n* = 33	32.0 (27.0–43.0)	22 (66.7)
PROMIS Upper Extremity (patient), *n* = 31	35.4 (28.5–44.9)	16 (51.6)
PROMIS Upper Extremity (parent), *n* = 28	23.5 (21.0–33.5)	20 (71.4)
**Pain:**		
Pain Intensity Now (patient), *n* = 48	1 (0–4)	–
Pain Intensity Now (parent), *n*-35	1 (0–3)	–
Pain Intensity Past 7 Days (patient), *n* = 48	3 (1–6)	–
Pain Intensity Past 7 Days (parent), *n* = 35	2 (0–6)	–
Pain Frequency, # Days in Past 14 Days (patient), *n* = 46	5 (1–13)	–
Pain Frequency, # Days in Past 14 Days (parent), *n* = 30	4 (0–14)	–
PROMIS Pain Interference (patient), *n* = 40	55.7 (50.3–61.4)	15 (37.5)
PROMIS Pain Interference (parent), *n* = 30	62 (51.5–66.5)	21 (77.8)

^a^Proportion of patients with a high symptom/low function based on total number of respondents

^b^Childhood Health Assessment Questionnaire

^c^Patient Reported Outcomes Measurement Information System

The median CHAQ score equaled 0.75, suggesting mild-to-moderate disability though CHAQ scores varied widely at the baseline visit (IQR: 0.030–1.875; min-max documented scores 0–3) [23]. PROMIS physical function measures suggested poor functional status for most participants with both Mobility (patient median (IQR): 36.9 (32.9–48.4); parent median (IQR): 32.0 (27.0–43.00)) and Upper Extremity Function (patient median (IQR): 35.4 (28.5–44.9); parent median (IQR): 23.5 (21–33.5)) domains significantly impacted; PROMIS physical function scores were often worse than 95% of the general pediatric population, with such poor scores noted in 39.6 and 66.7% of patient and parent Mobility assessments and 51.6 and 71.4% of patient and parent Upper Extremity assessments [22].

Pain intensity at the time of the study visit was modest (patient median (IQR): 1 (0–4); parent median (IQR): 1 (0–3), but patient pain intensity scores were higher when asked to average over a recall period of 7 days (patient median (IQR): 3 (1–6); parent median (IQR): 2 (0–6)). When estimating the number of days in which pain was experienced over the past 2 weeks, patient reported data showed somewhat higher frequency of pain (patient median (IQR): 5 (1–13)) compared to parent reported data (parent median (IQR): 4 (0–14)). While pain intensity was somewhat modest, PROMIS pain interference showed evidence of moderate to severe impact of pain on daily life (patient median (IQR): 55.7 (50.3–61.4); parent median (IQR): 62 (51.5–66.5); 37.5% of participants and 77.8% of parents reported pain interference scores that were worse than 95% of the general pediatric population [22].

## Discussion

The New CARRA Registry was able to enroll 119 new onset patients with Juvenile Dermatomyositis from 41 sites in North America in the first 12 months. This contemporary multicenter inception cohort of patients enrolling new onset JDM in North America will help to augment the knowledge from other North American and International cohorts [5, 10, 24–26].

In the first year, participants enrolled in the New CARRA Registry were similar in age and gender compared to other published series. Similar to other cohorts, over 90% of our cohort presented with characteristic JDM rash, namely Gottron’s rash or heliotrope rash as well as a variety of other JDM related skin manifestations including ulcerations and calcinosis. Calcinosis was uncommon at disease onset, occurring in 3% of the participants in our cohort, which is comparable to other cohorts early in the disease course [5, 27]. However, New CARRA Registry participants had lower baseline CHAQ and higher CMAS and MMT8 assessments suggesting less weakness at presentation compared to other published cohorts [27, 28] suggesting patients with milder disease activity were effectively enrolled, although this result could also be impacted by the inclusion of participants after treatment initiation. This conclusion is supported by the stratified subanalysis, where at enrollment, treatment-naïve participants had worse physician global scores and a trend for worse global skin scores and patient/parent global scores compared to treated participants. We plan additional subgroup analysis in the future. In addition, participants enrolled in the JDM New CARRA Registry had shorter median time to diagnosis compared to previous cohorts, suggesting possible improvement in present-day recognition and diagnosis of JDM. Similarly, severe disease features, including visceral organ involvement, were also uncommon in our present cohort.

Similar to the UK JDM study that reported 18% of their cohort presented without weakness [5], nearly 17 and 25% of JDM participants enrolled in the New CARRA Registry had no history of elevated muscle enzymes and proximal muscle weakness, respectively, suggesting a subset of participants that could be categorized as skin predominant or amyopathic JDM. Future subgroup analysis of treatments, longitudinal monitoring and long-term outcomes of this subset of participants will be of great interest to the pediatric rheumatology community. Familial aggregation of autoimmune disease has been hypothesized in JDM [29], especially lupus and type 1 diabetes. Similarly, family history of autoimmunity was reported in the JDM participants enrolled in the New CARRA Registry, including rheumatoid arthritis, psoriasis, lupus, and thyroid disease. Further analysis and consideration may be informative regarding disease pathogenesis and outcomes in this patient subset.

Patient−/parent-reported outcome measures (PROs) in this study demonstrated a substantial impact of JDM on multiple important domains. While patient/parent-rated disease activity was generally in the mild-moderate range [22], PROMIS PGH7 scores suggested only fair health status in the median JDM patient, with participants in the lowest quartile reporting health status worse than 95% of the general pediatric population [17]. Unsurprisingly, JDM participants experience significant loss of physical function early in their disease as reflected in PROs from this inception cohort, with different instruments demonstrating varying ability to detect functional limitations. Median CHAQ values fell in the mild-to-moderate disability range [23], though scores varied widely across the full possible range. PROMIS Mobility and Upper Extremity Function measures are normalized to data from large pediatric validation cohorts, providing an advantage in terms of interpretability. The median PROMIS Mobility and Upper Extremity Function scores in the New CARRA Registry were worse than 95% of the general pediatric population [23] suggesting very poor functional status early in the disease course that may be somewhat underestimated by the CHAQ. Pain intensity was generally modest, though pain may be intermittent as reflected in reports of pain occurring an average 4–5 of the past 14 days. While the intensity and frequency of pain were somewhat modest, the pain that JDM patients experience is highly disruptive, as reflected by PROMIS Pain Interference median scores in the moderate range for patient-report data and the severe range for parent-report data. Roughly one third of participants and three quarters of parents rated pain interference worse than 95% of the general pediatric population. These results suggest the substantial impact of pain on daily life that may not have been fully appreciated in prior literature in which pain intensity measures were solely used [23].

Myositis specific antibodies were assessed in about half of participants enrolled in the New CARRA Registry. The proportion of anti-NXP2 (22%) and anti-MDA5 (8%) antibodies is similar to what is reported in other cohorts, but there was lower prevalence of anti-TIF1-𝛄 (13%) and higher prevalence of anti-Mi-2 (11%) antibodies than other published cohorts [24, 30]. These differences in Myositis specific antibodies could contribute to the milder spectrum of disease found in this cohort. Similar to other cohorts, nearly 2/3 of participants had a positive ANA. As the Registry grows, we expect that additional studies related to autoantibody subtypes will be feasible to aid in future prognostication and advancement towards individualized treatment and precision medicine.

Over 40% of participants with moderate JDM were treated according to a CARRA JDM Consensus Treatment Plan demonstrating the potential for future analyses including comparative effectiveness research. The Registry is paired with a growing biorepository. Together we expect that these samples paired with well-phenotyped patient and disease characteristics from the Registry, will prove to be a rich resource in the future study of this rare condition.

As with any registry, there are several limitations to consider. Though this is an inception cohort of patients, participants were recruited voluntarily during routine clinical encounters. Therefore, it is possible that patients who had less severe disease activity were approached to participate and enrolled more often into the registry, while sicker patients may have been inadvertently excluded or less willing to enroll into the registry. Alternatively, participants could be enrolled at up to 12 weeks of treatment, so it is also possible that this cohort has milder disease features due to response to treatment during that time period. We had incomplete data disease measures important in the assessment of JDM, including VAS, MMT8, CMAS, and CDASI. The CHAQ respondent was not documented so patient/parent-reported data was pooled. Additional attention is necessary for accurate and consistent collection of PROs, according to age, for future data collection. These limitations are being addressed with additional education to CARRA Registry sites to improve future collection, in addition to data collection forms indicating when participants are unable to participate in strength testing due to age or disease severity, or in PRO collection due to age. In addition, there was low and heterogenous MSA reporting from CARRA sites, which is a limitation that is difficult to address, since MSA testing is not standardized to date and is performed through a variety of laboratories. There is current interest in systematically assessing MSAs in all JDM patients, including work to validate reliability of varying approaches to measuring MSAs, and a growing importance in how these measures may influence treatment strategies [30–32].

## Conclusions

The New CARRA Registry has been developed to improve the understanding of disease heterogeneity and to compare different treatments for rare pediatric rheumatic conditions including JDM. Nearly 120 JDM patients have been enrolled in its inaugural year. The collection of serial biosamples linked with the rich clinical, serologic and patient-reported data from this registry recorded longitudinally over the course of disease will allow for future novel studies and help to advance the science of JDM. This registry will also lay a foundation for comparative effectiveness research that will improve the treatment and outcomes of JDM patients in ways historical approaches were not able to achieve. We anticipate that such work will help improve patient outcomes in a real-life meaningful way.

## Data Availability

The data from this article were provided by the Childhood Arthritis and Registry Research Alliance (CARRA) by permission. Data may be shared on reasonable request to CARRA (carragroup.org).

## Abbreviations

CARRA: Childhood Arthritis and Rheumatology Research Alliance
CAT: Cutaneous Activity Tool
CDASI: Cutaneous Dermatomyositis Area and Severity Instrument
CHAQ: Childhood Health Assessment Questionnaire
CMAS: Childhood Myositis Assessment Scale
CTP: Consensus treatment plan
IQR: Interquartile range
JDM: Juvenile Dermatomyositisits
MDI: Myositis damage index
MMT8: Manual-Muscle Testing 8
MSA: Myositis Specific Antibody
PRO: Patient Reported Outcome
PROMIS**®**: Patient Reported Outcomes Measurement Information System
VAS: Visual Analog Score
